# SARS-CoV-2 epidemic in India: epidemiological features and *in silico *analysis of the effect of interventions

**DOI:** 10.12688/f1000research.23496.2

**Published:** 2020-06-29

**Authors:** Archisman Mazumder, Mehak Arora, Vishwesh Bharadiya, Parul Berry, Mudit Agarwal, Priyamadhaba Behera, Hemant Deepak Shewade, Ayush Lohiya, Mohak Gupta, Aditi Rao, Giridara Gopal Parameswaran

**Affiliations:** 1 All India Institute of Medical Sciences, New Delhi, Delhi, India; 2 All India Institute of Medical Sciences, Raebareli, Uttar Pradesh, India; 3 Operational Research, The Union South-East Asia Office, New Delhi, Delhi, India; 4 Centre For Operational Research, International Union Against Tuberculosis and Lung Disease (The Union), Paris, France; 5 Super Specialty Cancer Institute & Hospital, Lucknow, India

**Keywords:** COVID-19, Coronavirus, SIR model, India

## Abstract

**Background**: After SARS-CoV-2 set foot in India, the Government took a number of steps to limit the spread of the virus in the country. This included restricted testing, isolation, contact tracing and quarantine, and enforcement of a nation-wide lockdown starting 25 March 2020. The objectives of this study were to i) describe the age, gender distribution, and mortality among COVID-19 patients identified till 14 April 2020 and predict the range of contact rate; and ii) predict the number of  COVID-19 infections after 40 days of lockdown.

**Methods**: We used a cross-sectional descriptive design for the first objective and a susceptible-infected-removed model for *in silico* predictions. We collected data from government-controlled and crowdsourced websites.

**Results**: Studying age and gender parameters of 1161 Indian COVID-19 patients, the median age was 38 years (IQR, 27-52) with 20-39 year-old males being the most affected group. The number of affected patients were 854 (73.6%) men and 307 (26.4%) women. If the current contact rate continues (0.25-27), India may have 110460 to 220575 infected persons at the end of 40 days lockdown.

**Conclusion**: The disease is majorly affecting a younger age group in India. Interventions have been helpful in preventing the worst-case scenario in India but will be unable to prevent the spike in the number of cases.

## Introduction

Since December 2019, SARS-CoV-2, a novel virus of the *Coronaviridae* family of RNA viruses, has caused a widespread outbreak of the disease, now known as COVID-19, and was declared to be a pandemic by the World Health Organization (WHO) on March 11 2020 ^1– 3^. Human to human transmission occurs primarily through close-contact with the infected person, through fomites in the immediate surroundings of the infected person and via droplets of respiratory secretion, although there is limited evidence pointing to a possibility of airborne and faeco-oral transmission as well ^4– 7^. According to a few case studies, transmission may also occur via viral shedding in “pre-symptomatic” individuals during the incubation period ^8, 9^.

The incubation period for COVID-19 is thought to be within 14 days of exposure, with a median incubation period of 4–5 days ^4, 10, 11^. Globally, the median age of patients affected by COVID-19 is 47 years with the most common clinical findings being fever and cough ^4, 12^. About 18% of patients develop shortness of breath ^4^. Severe disease (including dyspnea defined as a respiratory rate of 30/min, blood oxygen saturation of 93%, a partial pressure of arterial oxygen to fraction of inspired oxygen ratio<300, and/or lung infiltrates>50% within 24 to 48 hours) has been reported to occur in 14% of elderly patients with pre-existing chronic diseases ^13^. Critical disease requiring intensive care unit admission has been reported in 5% of patients, and overall case-fatality rate is 2.3% ^13^. Currently, there are no approved treatments for COVID-19 and clinical trials, such as the WHO SOLIDARITY trial, are underway to evaluate the effectiveness of drugs like lopinavir-ritonavir, remdesivir, hydroxychloroquine and azithromycin ^3, 14^.

India reported its first case of COVID-19 on 30 January 2020; a medical student who had travelled from Wuhan, China, the then epicentre of COVID-19 ^15^. On 15th March 2020, India sealed its borders and stopped all international flights, meaning all initial imported cases in India arrived before 15 March 2020. According to the data available in the public domain, as of 12 April 2020, India had 8606 cases (both imported cases and due to person-to-person transmission). According to the Indian Council of Medical Research (the apex national medical research body), as of 31st March 2020, community transmission had not started then and India was in category 2 of WHO classification for transmission patterns, i.e. sporadic cases without evidence of community transmission ^16^. Recent evidence suggests 104 (1.8%) of the 5,911 patients with severe acute respiratory infection who tested positive for COVID-19, and 40 (39.2%) COVID-19 cases did not report any history of contact with a known case or international travel. This indicates imminent community transmission in the near future ^17^.

**Figure 1.**
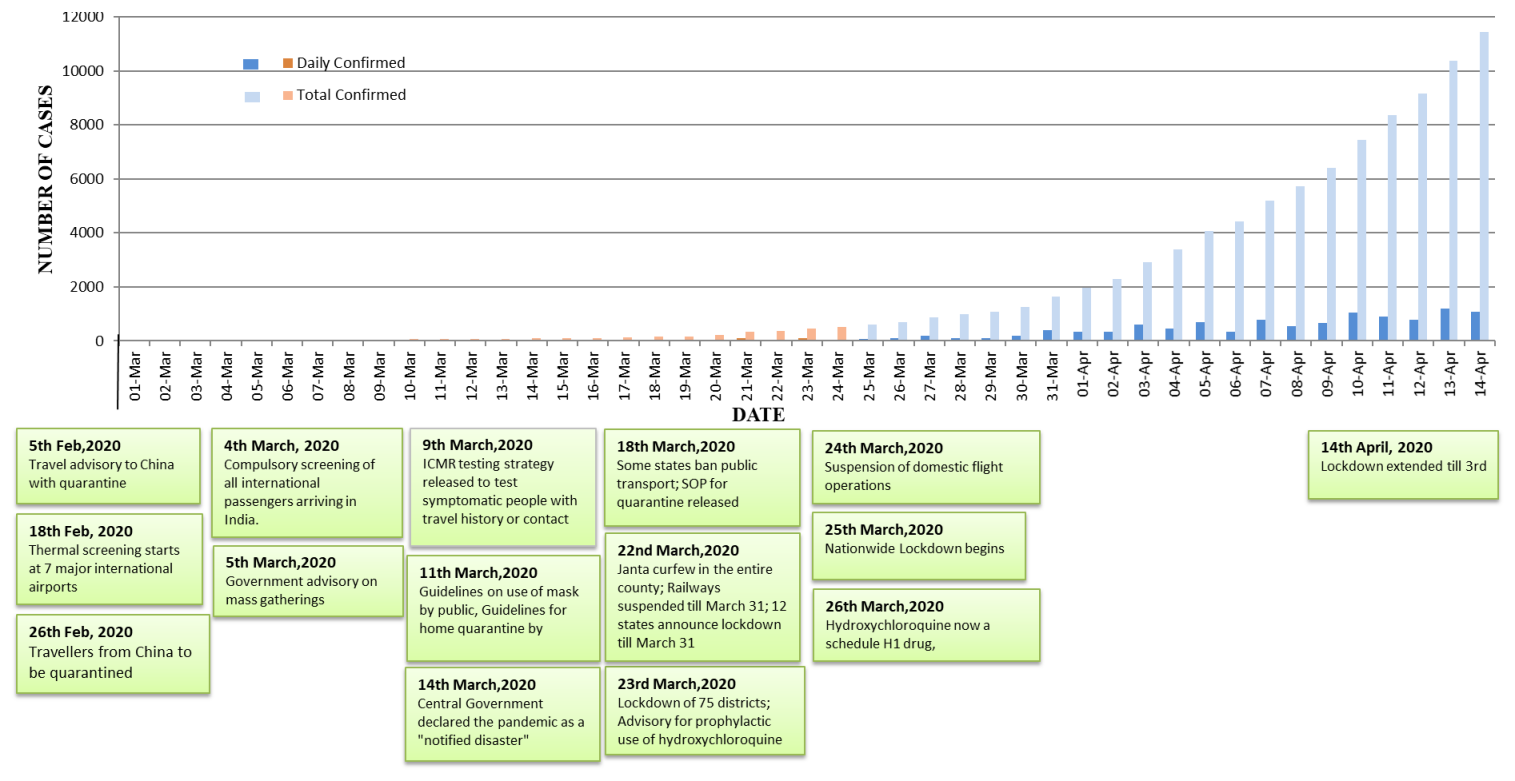
Cumulative cases of COVID-19 cases in India from 1st March 2020 to 14th April 2020. Data Source: ICMR-NIE and MoHFW ( www.mohfw.gov.in and www.covidindiaupdates.in). Orange, before lockdown; blue, after lockdown began.

Since the beginning of the outbreak in India, there have been a number of interventions done by both state level and central level governments ( Figure 1). These included restricting the inflow of international passengers, self-quarantine measures, directives on testing and management strategies, and in-country travel restrictions. During this period, the testing strategy also changed from being focused on foreign travel and contact history initially to including all individuals with severe acute respiratory illness and symptomatic health care providers. Gradually there were more social distancing measures in March, which were then followed by state-wise lockdowns, ultimately culminating in a nation-wide 21 day lockdown from 25 March 2020. As of 14th April, the lockdown had been extended until 3rd May 2020 (40 days).

In addition to the epidemiological parameters of COVID-19 in India, the other important question in the current scenario concerns the mathematical parameters of the initial spread of COVID-19 in India, and what are the epidemiological aspects that can predict this spread. We acknowledge there are certain difficulties in making precise calculations due to the rapidly changing dynamicity of the epidemic in the early stages, limited availability of data in the public domain, and limited testing capacity. Nonetheless, mathematical models with reasonable assumptions based on available information can help in analysis of the currently available data to provide important insights for guiding public health interventions ^18^. The most basic of these models is the susceptible-infected-removed (SIR) model ^19, 20^, which we have used in the current Indian scenario to determine the range in which contact rate β lies, calculate the range of the current reproduction number, Rt, and predict the number of COVID-19 infections at the end of the 40 day lock down period.

## Methods

### Epidemiological descriptive analysis of patients

This was a cross-sectional descriptive analysis of the laboratory confirmed COVID-19 patient-wise data collected from a crowdsourced database ( https://www.covid19india.org; includes data from state government and central government agencies). The data was taken for cases confirmed up to 14 April 2020, 7:20 PM Indian standard time or earlier.

Data analysis was done in regard to the age distribution, status of patients and gender distribution using Microsoft Office Excel 2007 (Microsoft, Redmond, WA, USA). Fatality rate in any category was found by dividing the number of deaths in the category by the number of affected individuals of that category.

### Mathematical modelling

The SIR model ^19, 20^ divides the (fixed) population of N individuals into three "compartments", which vary as a function of time (for purpose of this study, we have not included vital dynamics like birth and death rate because in COVID-19, the duration of infection is much less than the lifetime of an individual and hence they would not significantly affect the results):

- S(t) - S(t) are those susceptible but not yet infected with the disease (in a novel disease like COVID-19, the entire population is assumed to be susceptible as there is no pre-existing immunity);
- I(t) - I(t) is the number of infectious individuals;
- R(t) - R(t) are those individuals who have been removed from the infected population (includes those who have recovered from the disease and also deaths).

**Figure 2.**
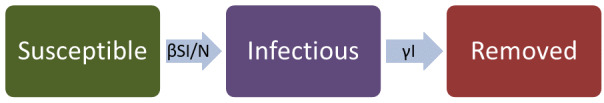
Flow diagram of transitions in susceptible-infected-removed (SIR) Model.

The SIR model describes the change in the population within each of these compartments in terms of two parameters, β and γ ( Figure 2). β describes the effective contact rate of the disease: a susceptible individual comes into contact with an infectious individual and acquires the infection. This parameter considers both the number of people contacted per unit time, and the effectiveness of transmission in each contact. It reflects the force of infection of the disease and helps us understand at what rate the epidemic is progressing. γ is the mean *removal rate*: in our model, it is calculated using the removed cases against new cases on a daily basis.

β and γ are useful in the SIR model using the following differential equations:

dS/dt=−βSI/N[1]

dI/dt=βSI/N−γI[2]

dR/dt=γI[3]

Comparing the equation from the SIR model and the general equation of exponential growth,

$$\beta =e^{r}+\gamma -1\;[4]$$

where r is the growth rate of the exponential curve. The Susceptible-Infectious-Removed-Susceptible (SIRS) model because reinfection has been rarely reported for COVID-19.

We have used the new cases of COVID-19 daily data available from 24th March to 13th April 2020, to estimate the two parameters (assuming a lag period of 11 days), β and R _t_ (time varying reproductive number) with the help of SIR model ( www.statista.com used for extraction of variables). We assumed that the recovery rate γ would remain constant for the population. The removal rate followed a normal distribution, and the mean was calculated with the data available from 1 March to April 4 2020 ^21^. Since the effect of interventions would reflect in the contact rate β, we then took the value of γ to be constant equal to the mean (0.103) and ran the SIR model multiple times by varying the value of β, and comparing the trends with the real data. We assumed uniform transmission for all the simulations.

We plotted the trend line for the real data using Microsoft Office Excel 2007, and used the equation of the curve to find out the trend of β in India in the present-day scenario by comparing it to equation 4. We then used the present trends of β to estimate the expected number of infections at the end of 40 days lockdown.

### Ethics

Anonymized data available data in the public domain was used for analysis. Ethical approval was not required.

## Results

### Epidemiological descriptive analysis of patients

There were 10939 total patients recorded; age and gender data were available for 1161 (10.61%). Of 1161 patients, 854 (73.6%) were men and 307 (26.4%) were women. Median age was 38 years (IQR, 27–52). Nearly half of the patients (43.2%) were in the age group of 20–39 years. The median age of women was 40 years (IQR, 24–56.5) and men was 38 years (IQR, 27–51) ( Table 1).

**Table 1.** Distribution of COVID-19 patients in India confirmed until 14 April 2020 across different age groups and gender (n=1161).

	Men		Women	
Age group	N	%(normalized value) *	N	%(normalized value) *
<20years	66	5.68(0.31)	43	3.70(0.22)
20–39 years	391	33.68(1.94)	110	9.47(0.61)
40–59 years	274	23.60(2.13)	86	7.41(0.70)
60–79 years	116	9.99(2.20)	63	5.43(1.17)
>=80 years	7	0.60(1.40)	5	0.43(0.81)
**Total**	**854**		**307**	

*Normalized values have been calculated by dividing the percentage of patients in each category by the percentage of Indian population in that category.

Out of 1161 patients, 1038 hospitalizations and 29 deaths were documented. The majority of them (65.5%) were in the 60–79 year age group. The median age of deceased patients was 67 years (IQR, 57–71). The mortality rate in male patients was 2.3% and for female patients was 2.9%. Overall case fatality was 2.5%. There was a 10.6% mortality in the 60–79 years age group and 16.7% in individuals aged equal to or above 80 years ( Table 2).

**Table 2.** Distribution of COVID-19 patients of India with age, gender and status of disease updated till 14 April 2020 (n=1161).

Variables	Deceased	Recovered	Hospitalized *
AGE			
*<20years*	1	8	100
*20–39 years*	2	47	452
*40–59 years*	5	22	333
*60–79 years*	19	14	146
*>=80 years*	2	3	7
GENDER			
*Male*	20	66	768
*Female*	9	28	270
**TOTAL**	**29**	**94**	**1038**

*Hospitalized include patients in facility isolation, in ICU or under medical care in hospital

In total, 94 patients had recovered. Among the recovered patients, 47 (50.0%) were in the 20–39 year age group, 22(23.4%) in the 40–59 year age group. Median age for recovered patients was 35 years (IQR, 24–53). 

### Mathematical modelling

**Figure 3.**
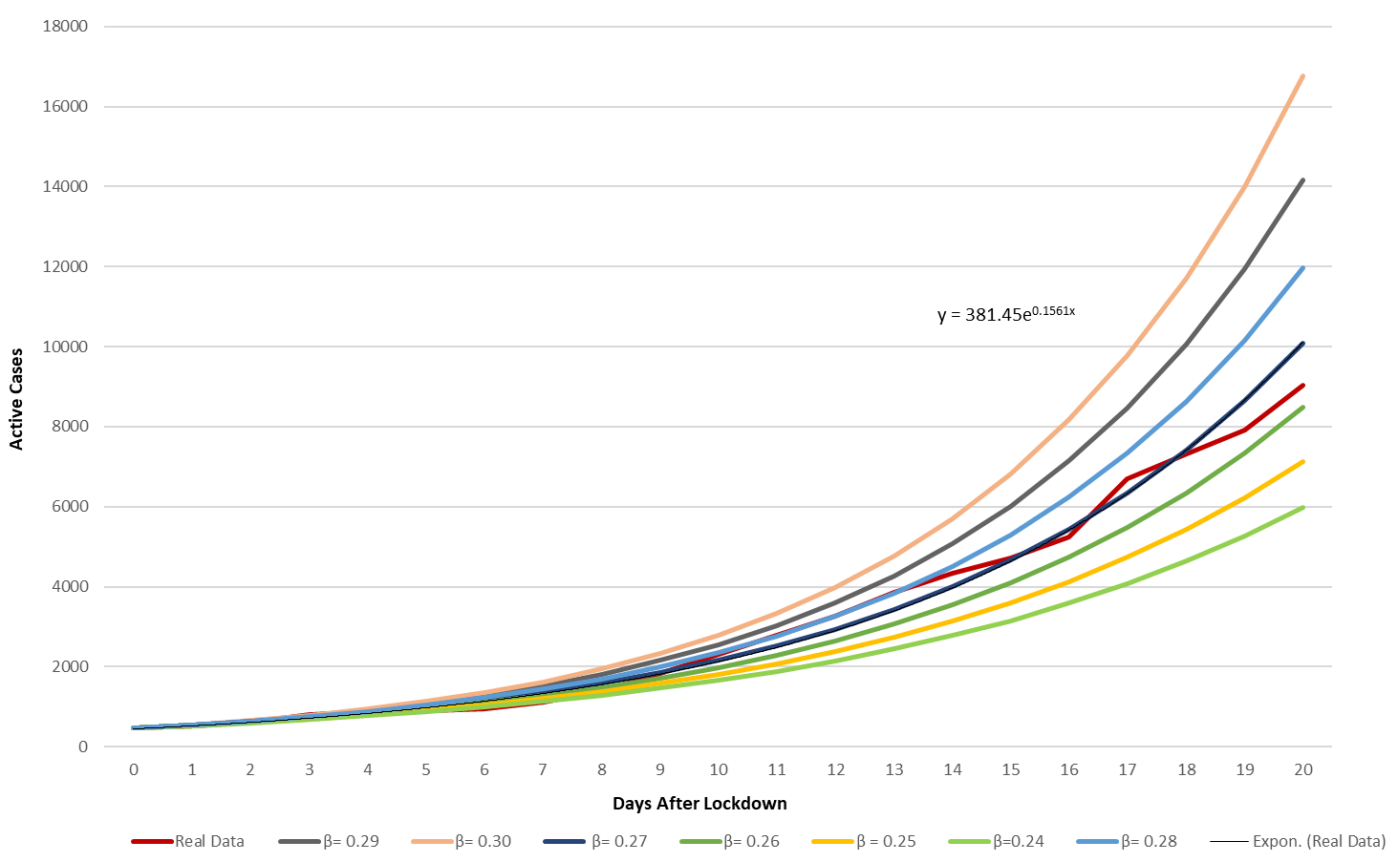
Estimation of β from Indian data. Datasource: https://www.statista.com/statistics/1104054/india-coronavirus-covid-19-daily-confirmed-recovered-death-cases/.

After running multiple simulations using the SIR model, all assuming different values of β we found that the value of β in the current Indian scenario calculated from the trendline of real data lies around 0.272, which is also visible in the graph of real-time active cases lying between β=0.25 and β=0.28 curves ( Figure 3).

Also visible in the graph, is the real data line shifting from 0.24 to 0.28 from day 8. Also, as R _t_= β/ **γ**, the value of the reproductive number is found to be varying between 2.5–2.8 in the Indian scenario until 13th April 2020. Considering the present trend of β, the number of infected persons after the 40 day lockdown period may vary from 110,460 to 220,575.

## Discussion

To the best of our knowledge, this is the first study to predict the number of COVID-19 infected persons in India after the 40 days of lockdown. We have used crowd sourced data due to lack of availability of official data. We could not include all the cases in the epidemiological analysis due to unavailability of the demographic details of all the patients. Limitations notwithstanding, there were three key findings.

First, the median age of affected individuals is lower than reported in other countries. In study cohorts of Wuhan, the median age of affected patients ranges from 49–56 years ^4, 22, 23^. In previous studies on COVID-19, it has been established that risk increases with age and comorbidities ^13, 22, 23^. The broad based nature of India’s population pyramid means there are more people in the younger age group and very few people in the ≥80 years age group. Hence, because of more percentage of the population in the younger age group, they are more likely to be infected. Another reason is that as there is limited evidence of community spread in the epidemic in India so far; it has been reported to be driven by imported cases who mostly belong to the younger age group. In 2018, Indian residents between 35 and 49 years of age took the most holidays outside the nation ^24^. Hence, in the early part of the epidemic, younger people would be more affected as they would constitute more of the imported cases. On normalizing the percentage of patients in each age group with the corresponding percentage representation of the population as shown in Table 1, we observed that the highest normalized ratio of number of patients is in the 60–79 years age group category. Interestingly, according to this analysis, men in the 60–79 years age group are affected more than the ≥80 years age group. This is something that has not been reported until now and it has to be seen whether this changes as the number of cases in India grows. This is new to the literature of COVID-19 and needs to be studied further.

Second, we found a case fatality rate of 2.5%, which is lower than countries like Italy ^25^. The reason for this could be that Italy is in the later phases of the epidemic with widespread community transmission and high mortality due to overburdening of the health system. This has also led to focussing testing on severe patients and possible advise to suspected patients with mild symptoms to stay at home. In China, the case fatality rate was found as 2.3%, 14.8% in the above 80 years population, and 8.0% in the 70–79 years population ^13^. Our estimate gives a slightly higher value, which may be due a smaller sample size or can also be because mild cases of COVID-19 have so far been missed due to restricted testing strategy during the initial stage.

Third, from the SIR model, the values of β and the trend for R _t _shows that the interventions which were put in place by the Indian government starting from mid-March were partially effective, preventing the scenario where R _t_ can reach even more than four ^26^. The increase in β from day eight is probably due to the identification of a cluster in Delhi ^27^. Additionally, Indian Council of Medical Research recently updated the testing strategy, which might have increased the detection of the positive cases significantly. According to a study in the early days of the epidemic, Wuhan city and Hubei province reported R _t_ between 1.85 and 4.46, which aligns with our study findings ^26^. All over China, the R _t_ varied from 1.23 to 5.77. South Korea, which has high population density like India, had a decreasing trend of R _t_ from 9.72 on 20 February to 1.50 on 7 March. This indicates that the interventions have been helpful in preventing the worst case scenario in India but is unable to prevent the spike in number of cases ^27, 28^.

At the end of the 40 days lockdown, with the range of β at the time of study, India is likely to have a significant number of infected persons (110,460 to 220,575) that would include both symptomatic and asymptomatic individuals. It may also enter the exponential growth phase and it would become very difficult to contain at that stage. The situation can still be controlled if R _t_ can be brought down close to one. This indicates the need for more effective strategies and ensuring optimum testing to avoid underestimation of danger. However, it has to be noted that our study does not predict the β for India in the future, rather we extrapolated based on the range at the time of study.

In a modeling study of the Indian scenario, it was shown that India will have significant number of cases without intervention with widespread screening such as in airports delaying the epidemic and quarantine would slow local spread, decreasing the cumulative incidence by 2% to 62% ^29^. Our study also shows how without decreasing R _t_, India will have very high number of cases. Modeling studies have also been done that show that as the lockdown is eased, the testing should be increased to control the epidemic in India effectively, and the easing should be a gradual process and if lockdown is effective the peak prevalence can be decreased to a large extent ^30^. 

Future research is needed to support the current findings that unlike other countries, in India, COVID-19 is mostly affecting the 20–39 years age group since our data analysis was restricted to individuals in whom data was available. This study was conducted using data from the initial part of the epidemic in India. The SIR model used does not account for age-structure or comorbidities. Hence we are addressing more dynamicity and realness by taking into account multiple compartments with robust mathematical assumptions in our future models to study the current pandemic. Comprehensive studies based on the clinical manifestations and laboratory parameters including genomic sequencing to detect mutations of the virus also needs to be done for the Indian population to see if the clinical features and the viral strains differ from other populations.

To conclude, the COVID-19 epidemic in India is more affecting younger age groups as compared to other countries. Our mathematical model predicts that India will have a significant number of infected persons after the 40 days lockdown. Hence, strict social distancing, and optimum testing followed by isolation and quarantine are vital elements to control the COVID-19 epidemic.

## Data availability

### Underlying data

Raw data for daily cases of COVID-19 with gender, age and patient status obtained from: patientdb.covid19india.org

Raw data for estimation of β obtained from: https://www.statista.com/statistics/1104054/india-coronavirus-covid-19-daily-confirmed-recovered-death-cases/

Figshare:Mazumdaer A *et al.* study - COVID 19 India data, https://doi.org/10.6084/m9.figshare.c.4940472.v2 ^31^.

This project contains the following data:

- Confirmed daily cases of COVID-19
- Full patient dataset COVID-19
- Indian population data

Data are available under the terms of the Creative Commons Zero "No rights reserved" data waiver (CC0 1.0 Public domain dedication).
