# Supplementary material for: SARS-CoV-2 epidemic in India: epidemiological features and in silico analysis of the effect of interventions
Source: F1000Res. 2020 Jun 29;9:315. Originally published 2020 Apr 30. [Version 2] doi: 10.12688/f1000research.23496.2 (PMC7262570; doi:10.12688/f1000research.23496.2)
Supplement: Peer review history

# Peer review: Reviewer response for version 1

**DOI:** 10.5256/f1000research.25930.r63081

**Published:** 2020-05-29

**Authors:** Soumya Swaroop Sahoo

**Affiliations:** 1 Department of Community & Family Medicine, All India Institute of Medical Sciences, Bathinda, Punjab, India

A very good attempt to present the Indian COVID-19 scenario by the authors. I congratulate them on their work.

 However a few queries:

1. The data analysis has been performed on 1161 patients. To project it for such a large population has limited scope.
2. The range of the projection of 1.10 lakh to 2.20 lakh is quite wide. How do we interpret it?
3. In COVID, most of the patients recover in due course. If possible, the SEIR model could have been used for a better picture.
4. The discussion section contains a comparison with the descriptive data of different countries. It could have been compared with the modeling data of different studies of other countries to highlight the similarity/difference of the Indian scenario.
5. The authors in the discussion state that "we observed that the highest number of patients is in the 60–79 years age group category. The exact percentage projected needs to be mentioned.
6. The assumptions that the authors took for the modeling approach needs to be clearly stated in the methodology apart from birth and death rate.

Is the work clearly and accurately presented and does it cite the current literature?

Yes

If applicable, is the statistical analysis and its interpretation appropriate?

Partly

Are all the source data underlying the results available to ensure full reproducibility?

Yes

Is the study design appropriate and is the work technically sound?

Yes

Are the conclusions drawn adequately supported by the results?

Yes

Are sufficient details of methods and analysis provided to allow replication by others?

Yes

Reviewer Expertise:

Community Medicine and Epidemiology

I confirm that I have read this submission and believe that I have an appropriate level of expertise to confirm that it is of an acceptable scientific standard.

# Response

**Published:** 2020-06-12

**Authors:** PRIYAMADHABA BEHERA

**Affiliations:** All India Institute of Medical Sciences, Raebareli, India

Reply to Dr. Palepu’s (Reviewer 1) comments:

**Comment 1**: The mathematical model used is "SIR", standing for "Susceptible, Infectious, Recovered/dead". This needs modification throughout the manuscript.

**Author Response 1**: We have used the full form as Susceptible-Infectious-Removed model, where removed includes Deceased and Recovered. Thank you.

**Comment 2**: In COVID-19, re-infection is being seen in a few patients. This can be explained in limitation as to why the "SIRS" model was not considered over "SIR". And also, not all susceptible individuals are infected. Why was the "SEIR" model not considered by authors?

**Author Response 2**: We have added the reason for not using the SIRS model in the methods section. We didn’t consider the SEIR model because, at the time when this study was done, sufficient data was not available in the public domain for a reliable estimate of alpha for India which is the rate of transition from the exposed pool to the infected pool. In view of the limitations in this model, we have developed another model that is available at https://doi.org/10.1101/2020.05.13.20096826. Thank you.

**Comment 3**: Although, the model mentions that the birth rate and death rate were not taken into account, its effect on outcome estimation has not been explicitly mentioned. The reason that the duration of infection is much lesser than the lifetime of an individual can be explanatory for non-inclusion of birth and death rate.

**Author Response 3**: We have added the reason for the non-inclusion of vital dynamics in the methods section and how their inclusion would not significantly affect the results. Thank you.

**Comment 4**: Clear mention of the force of infection with proper notation is required.

**Author Response 4**: For the study, we have calculated gamma and tried to compare the real trends with trends for the varying value of beta. For this reason, we have not clearly mentioned the force of infection as it would not be reliably derived from our study. Also, because of varying R _t_, it would be difficult to give a definite value for the force of infection in India. Thank you.

**Comment 5**: Reasons for the younger age group being affected more can be explained in greater detail.

**Author Response 5**: We have explained possible reasons like a high percentage of the population in the younger age group in India and possible younger age of imported cases in more detail in the discussion. Thank you.

**Comment 6**: Various studies have been published to date with different modeling techniques for the estimation of COVID-19 cases. They can be used for comparison in the discussion section.

**Author Response 6**: We have added discussions about other modeling studies of India in the discussion section.  Thank you. When we were doing the study, the epidemic had progressed in other countries, so we compared our data with the actual data of the other countries. Also, the aim of our study was not to exactly predict the number of cases but to see the trend of the real data and get an idea about parameters like R _t_, and contact rate. We extrapolated to give an idea of the number of cases India could have reached if the R _t_ had not been decreased by strict interventions. It must be noted that our study does not predict the future trend of beta, rather extrapolation has been done based on the range of beta in India till the time of the study.

# Response

**Published:** 2020-06-12

**Authors:** PRIYAMADHABA BEHERA

**Affiliations:** All India Institute of Medical Sciences, Raebareli, India

Reply to Dr. Sahoo’s (Reviewer 2) comments:

**Comment 1**: The data analysis has been performed on 1161 patients. To project it for such a large population has limited scope.

**Author Response 1**: Thanks for your valuable comments. The data for 1161 patients were used for the study of the epidemiological features of the affected people in India. For the model, the real data of the number of active cases was plotted. However, we agree that using data from the initial stage of the epidemic in India is one of our limitations, and this has been included in the discussion. This study was done to provide an overview of what India might be dealing with.

**Comment 2**: The range of the projection of 1.10 lakh to 2.20 lakh is quite wide. How do we interpret it?

**Author Response 2**: We have incorporated the explanation for this wide range of finding in the discussion. With the preliminary data available in the public domain, we obtained this interval based on the range of beta at the time of the study. Our study does not predict the beta for India in the future, rather we extrapolated based on the range at the time of the study. The range gives an idea of the number of infections if the contact rate wasn’t affected by lockdown and strict social distancing. Thank you.

**Comment 3**: In COVID, most of the patients recover in due course. If possible, the SEIR model could have been used for a better picture.

**Author Response**: As we have mentioned in the reply to Dr. Palepu’s 2 ^nd^ comment, we didn’t consider SEIR model because, at the time when this study was done, sufficient data was not available in the public domain for a reliable estimate of alpha for India which is the rate of transition from the exposed pool to the infected pool. In view of the limitations in this model, we have developed another model that is available at https://doi.org/10.1101/2020.05.13.20096826. Thank you.

**Comment 4**: The discussion section contains a comparison with the descriptive data of different countries. It could have been compared with the modeling data of different studies of other countries to highlight the similarity/difference of the Indian scenario.

**Author Response 4**: When we were doing the study, the epidemic had progressed in other countries, so we found it better to compare our data with the actual data of the other countries. We have modified our discussion including modeling studies form the Indian scenario. Thank you.

**Comment 5**: The authors in the discussion state that "we observed that the highest number of patients is in the 60–79 years age group category. The exact percentage projected needs to be mentioned.

**Author Response 5**: This observation is based on the normalized values calculated and tabulated in Table 1. The language has been modified for clarity. Thank you.

**Comment 6**: The assumptions that the authors took for the modeling approach needs to be clearly stated in the methodology apart from birth and death rate.

**Author Response 6**: Apart from the assumption regarding birth and death rate, we have assumed the value of removal rate gamma to be constant as we have mentioned in the text. We assume uniform transmission for all our extrapolations. These have been described in the method section. Thank you.

# Peer review: Reviewer response for version 1

**DOI:** 10.5256/f1000research.25930.r63079

**Published:** 2020-05-22

**Authors:** Sarika Palepu

**Affiliations:** 1 Department of Community and Family Medicine, Vir Chandra Singh Garhwali Government Institute of Medical Sciences and Research, Srinagar, Uttarakhand, India

1. The mathematical model used is "SIR", standing for "Susceptible, Infectious, Recovered/dead". This needs modification throughout the manuscript.
2. In COVID-19, re-infection is being seen in a few patients. This can be explained in limitation as to why "SIRS" model was not considered over "SIR". And also, not all susceptible individuals are infected. Why was the "SEIR" model not considered by authors?
3. Although, the model mentions that the birth rate and death rate were not taken into account, its effect on outcome estimation has not been explicitly mentioned. The reason that the duration of infection is much lesser than the lifetime of an individual can be explanatory for non-inclusion of birth and death rate.
4. Clear mention of the force of infection with proper notation is required.
5. Reasons for the younger age group being affected more can be explained in greater detail.
6. Various studies have been published to date with different modelling techniques for the estimation of COVID-19 cases. They can be used for comparison in the discussion section.

Is the work clearly and accurately presented and does it cite the current literature?

Yes

If applicable, is the statistical analysis and its interpretation appropriate?

Partly

Are all the source data underlying the results available to ensure full reproducibility?

Yes

Is the study design appropriate and is the work technically sound?

Yes

Are the conclusions drawn adequately supported by the results?

Yes

Are sufficient details of methods and analysis provided to allow replication by others?

Yes

Reviewer Expertise:

Public health and Epidemiology of infectious diseases

I confirm that I have read this submission and believe that I have an appropriate level of expertise to confirm that it is of an acceptable scientific standard.
